# The Triglycerides and Glucose (TyG) Index Is Associated with 1-Hour Glucose Levels during an OGTT

**DOI:** 10.3390/ijerph20010787

**Published:** 2022-12-31

**Authors:** Mattia Massimino, Giuseppe Monea, Giuseppe Marinaro, Mariangela Rubino, Elettra Mancuso, Gaia Chiara Mannino, Francesco Andreozzi

**Affiliations:** 1 Department of Medical and Surgical Sciences, University Magna Graecia of Catanzaro, 88100 Catanzaro, Italy; 2 Research Center for the Prevention and Treatment of Metabolic Diseases (CR METDIS), University Magna Grecia of Catanzaro, 88100 Catanzaro, Italy

**Keywords:** prediabetes, 1 h post-load glucose, triglyceride–glucose index, impaired glucose tolerance, insulin resistance

## Abstract

Background and Objectives: Among individuals with normal glucose tolerance (NGT), subjects with high levels of plasma glucose (≥155 mg/dL) at sixty minutes during an oral glucose tolerance test (1h-OGTT) are at an increased risk of developing type 2 diabetes. We investigated the association between the triglycerides and glucose (TyG) index, a novel marker of insulin resistance, with 1h-OGTT glucose plasma concentrations. Material and Methods: 1474 non-diabetic Caucasian subjects underwent a 75 g OGTT and were divided into two groups according to the cutoff 1h-OGTT plasma glucose < 155 mg/dL (NGT-1h-low) and ≥ 155 mg/dL (NGT-1h-high). The TyG index was calculated as ln [fasting triglycerides (milligrams per deciliter) × fasting blood glucose (milligrams per deciliter)/2]. Multivariable linear and logistic regression analyses were used to establish the contribution of the TyG index to the variability of 1h-OGTT glucose, and how the former affected the risk of being NGT-1h-high. Results: 1004 individuals were NGT-1h-low and 470 were NGT-1h-high. The TyG index was higher for NGT-1h-high (*p* = 0.001) individuals, and it was an independent factor influencing 1h-OGTT glycemia (β = 0.191, *p* < 0.001) after correcting for age, sex, and BMI. The TyG index was the strongest marker associated with the risk of being NGT-1h-high (OR = 1.703, CI 95% 1.34–2.17, *p* < 0.001) when compared with FPG (OR = 1.054, CI 95% 1.04–1.07, *p* < 0.001) and the HOMA-IR (OR = 1.156, CI 95% 1.08–1.23, *p* < 0.001). Conclusions: Our study demonstrated that the TyG index, an efficient and cost-effective marker of insulin resistance, is associated with the variability of early post-challenge glucose levels and is an independent marker of being NGT-1h-high.

## 1. Introduction

Type 2 diabetes mellitus (T2DM) is a chronic metabolic disorder characterized by persistent hyperglycemia due to impaired insulin secretion, peripheral tissue resistance to insulin action, or a combination of both [1]. Data from 2021 allows to estimate that 10.5% of 20–79 year-olds (536.6 million people) were affected by diabetes, expected to raise to 12.2% in 2045 (783.2 million people), with a large impact on health and social expenditures [2].

Currently, the adoption of pharmacological therapies alongside lifestyle interventions have proven to be capable of reducing the incidence and progression of macro- and microvascular-T2DM-linked complications [3,4]. In the field of prevention, a key aspect is the detection of high-risk subjects. Much evidence has already shown that the presence of high levels of plasma glucose at 1-hour post-load during an oral glucose tolerance test (OGTT) identifies a new category of individuals at an increased risk of developing T2DM among subjects with otherwise normal glucose tolerance (NGT) [5,6,7]. Indeed, the value of 1-hour post-load glucose levels during an OGTT (1h-OGTT) was revealed to be a better predictor of dysglycemia among individuals without T2DM than HbA1c 2 h post-load plasma glucose during an OGTT and fasting plasma glucose (FPG) [8,9,10,11,12,13,14]. Moreover, it has now been established that normoglycemic subjects with a 1h-OGTT plasma glucose ≥ 155 mg/dL (NGT-1h-high) have higher incidence rates of diabetes complications, cardiovascular risks, and mortality [14,15,16]. This being the case, the detection of effective biomarkers (possibly non-invasive ones) able to refine the stratification of naive, prediabetic or 1-hour-high patients, could be useful for the design of tailored strategies for preventing and/or delaying macro- and microvascular complications that shorten life expectancy and reduce life quality in those patients [16]. 

Several studies have reported that non-insulin-based indices of insulin resistance could be useful tools for the early detection of insulin resistance [17,18]. Among the several indices available in the literature, our attention is focused on the “the TyG index” (triglycerides and glucose index), which takes into account fasting triglycerides and fasting plasma glucose levels [19,20,21]. Most intriguingly, the TyG index (a non-insulin-based index) has been demonstrated to have a good closeness of agreement to the gold standard, the hyperinsulinemic–euglycemic clamp, in estimating the severity of insulin-resistance and to be equal—or even superior—to the HOMA-IR index (homeostatic model assessment of insulin resistance) [17,22].

The aim of our cohort study was to investigate whether the TyG index was associated with 1h-OGTT glucose plasma concentrations in a large cohort of 1474 Caucasian non-diabetic adult individuals participating in the Catanzaro metabolic risk factors (CATAMERI) study.

## 2. Materials and Methods

### 2.1. Study Population

The study group consisted of 1474 self-reported non-diabetic Caucasian participants (661 men and 813 women, aged 18–83, mean age 46.01 ± 13.202), enrolled in the Catanzaro metabolic risk factors study (CATAMERIS), an observational study assessing cardiometabolic risk factors in individuals carrying at least one risk factor, including overweight/obesity, hypertension, dyslipidemia, dysglycemia, and a family history of type 2 diabetes [23,24].

The exclusion criteria were the presence of autoimmune diabetes, a diagnosis of T2DM, current or former therapy with hypoglycemic agents, chronic gastrointestinal diseases, chronic pancreatitis, a history of any malignant disease, a history of alcohol or drug abuse, liver or kidney failure, and treatments able to modulate glucose metabolism, including lipid-lowering and hypoglycemic agents. During the first visit in the morning after a 12 h fast, anthropometrical parameters, such as body mass index (BMI), systolic (SBP), and diastolic (DBP) blood pressure, were measured, and blood samples were collected for the assessment of biochemical parameters, including triglycerides levels. Height was measured to the nearest 0.1 cm, while body weight was measured with a calibrated electronic scale to the nearest 0.1 kg. BMI was calculated as body weight in kilograms divided by height in square meters (kg/m^2^). A 75 g oral glucose tolerance test (OGTT) was performed with 0, 30, 60, 90, and 120 min samplings for circulating plasma glucose and insulin measurements. Glucose tolerance statuses were evaluated according to World Health Organization (WHO) criteria [25]. Individuals were thus classified as having:

- NGT when their fasting plasma glucose (FPG) was <110 mg/dL (6.1 mmol/L) and their 2 h post-load glucose was <140 mg/dL (7.77 mmol/L);
- IFG (impaired fasting glucose) when their FPG was 110–125 mg/dL (6.1–6.9 mmol/L) and their 2 h post-load glucose was <140 mg/dL (7.77 mmol/L);
- IGT (impaired glucose tolerance) when their FPG was <126 mg/dL (7 mmol/L) and their 2 h post-load glucose was 140–199 mg/dL (7.77–11.0 mmol/L).

Only individuals classified as having NGT were considered for the present report. The study was approved by the institutional ethics committee of the university “Magna Graecia” of Catanzaro (approval code: 2012.63). Written informed consent was obtained from each participant in accordance with the principles of the Declaration of Helsinki.

### 2.2. Laboratory Determinations

Glucose, triglycerides, and total and high-density lipoprotein (HDL) cholesterol concentrations were determined by enzymatic methods (Roche, Basel, Switzerland). The plasma insulin concentration was measured with a chemiluminescence-based assay (Immulite, Siemens Healthcare Diagnostics Inc., Marburg, Germany).

### 2.3. Calculations

Participants with normal glucose tolerance (NGT) were divided into two groups according to their 1 h plasma glucose concentrations during the OGTT. Those with a 1 h plasma glucose equal or above 155 mg/dL (8.6 mmol/L) were labeled NGT-1h-high; those with a 1 h plasma glucose below 155 mg/dL were defined as NGT-1h-low. The homeostasis model assessment (HOMA) index was calculated as fasting insulin × fasting glucose/22.5 [26]. The triglycerides–glucose (TyG) index was calculated as ln (fasting triglycerides [milligrams per deciliter] × fasting glucose [milligrams per deciliter]/2) [27].

### 2.4. Statistical Analysis

The results for the continuous variables are given as means ± SD. Anthropometric and metabolic differences between groups were tested after adjusting for age, gender, and BMI using a general linear model. The χ2 test was used for the comparisons of categorical variables. Variables with a skewed distribution (i.e., triglycerides, fasting, 30 min, 90 min, 1 h, and 2 h insulin, and the HOMA index) were log-transformed to meet the normality assumption for statistical purposes. A multivariable stepwise linear regression was used to examine the relation of 1h-OGTT glucose and the TyG index in a statistical model including several potential confounders: age, gender, BMI, fasting glucose levels, and the HOMA index. Similarly, a multivariable logistic regression model was computed in order to evaluate the risk of falling into the NGT-1h-low or NGT-1h-high groups. The Bonferroni post hoc correction for multiple comparisons was applied. The receiver–operating characteristic (ROC) analysis was used to assess the predictive value of the TyG index (area under the curve). Youden’s J statistic was calculated to identify the optimal cutoff value of the TyG index for discriminating between the NGT-1h-low and NGT-1h-high groups (i.e., the value that maximizes the difference between the true positive and false positive rates of classification).

A *p* value < 0.05 was considered statistically significant. All analyses were performed using the statistical package SPSS 22.0 for Windows (SPSS, IBM, Chicago, IL, USA).

## 3. Results

The anthropometric and biochemical characteristics of the study cohort are summarized in Table 1. A total of 1474 non-diabetic participants with normal glucose tolerance (NGT) were evaluated and divided into two groups using a 1h-OGTT plasma glucose cutoff point of 155 mg/dL: 1004 (68%) participants with a 1h-OGTT plasma glucose < 155 mg/dL (NGT 1h -low) and 470 (32%) individuals with a 1h-OGTT plasma glucose ≥ 155 mg/dL (NGT-1h-high). Gender, age, and BMI distributions were unevenly scattered between the two groups. The NGT-1h-low group harbored more men than women, while the NGT-1h-high participants were older and exhibited higher glycemic values compared with the NGT-1h-low participants. Because these parameters were associated with glycemic control, all subsequent analyses were adjusted for age, gender, and BMI. After adjusting for age, gender, and BMI, the NGT-1h-high group exhibited significantly higher fasting, 2h-OGTT glucose, and insulin levels compared with the NGT-1h-low group. Additionally, the triglycerides levels and the TyG index were significantly higher in the NGT-1h-high group compared with the NGT-1h-low individuals, while no significant differences were observed between the groups for total cholesterol levels (Table 1).

To evaluate the independent factors influencing the variability of 1h-OGTT plasma glucose, a multivariate linear stepwise regression analysis was run in four models including gender, age, and BMI as covariates (Table 2). Model 1 additionally included the TyG index, resulting in a significant association with 1h-OGTT (β = 0.191; *p* < 0.001; model-adjusted R^2^ = 21.6%). In Model 2, FPG was inserted instead of the TyG index, and this resulted in a consistent association with 1h-OGTT glucose levels (β = 0.284; *p* < 0.001; model-adjusted R^2^ = 25.3%). Model 3 evaluated the association of 1h-OGTT glucose levels with the HOMA-IR, the result of which was weaker than that with the TyG index (β = 0.159; *p* < 0.001; model-adjusted R^2^ = 20.5%). Because the TyG index was calculated from fasting glucose measurements, we wondered whether the effects exerted by TyG on the early post-challenge glucose levels were merely a consequence of the FPG component of the formula. To assess this issue, we performed a sensitivity analysis in Model 4 by including the TyG index and FPG together as co-factors. The two variables remained significantly associated with 1h-OGTT; in particular, FPG showed a stronger association (β = 0.249; *p* < 0.001) compared with the TyG index (β = 0.115; *p* = 0.032; model-adjusted R^2^ = 26.3%). Altogether, our models suggested that the TyG index was an independent marker of the variability of 1h-OGTT glucose levels, even if the association was partially attributable to FPG. 

To estimate the independent contribution of the TyG index to the risk of falling in the NGT-1h-high group, we performed a multiple logistic regression analysis, summarized in Table 3. Models 1 to 4 included age, gender, and BMI as covariates. In the first model (Model 1), the TyG index was additionally included as an independent variable, resulting in a significant association with increased odds of being NGT-1h-high (OR = 1.703, CI 95% 1.34–2.17, *p* < 0.001). In Model 2, the independent contribution of FPG was evaluated; as expected, it was significantly associated with being NGT-1h-high (OR = 1.054, CI 95% 1.04–1.07, *p* < 0.001). Model 3 evaluated the contribution of th HOMA-IR, resulting in a significant association with a higher risk of being NGT-1h-high (OR = 1.156, CI 95% 1.08–1.23, *p* < 0.001). The final model (Model 4) included both the TyG index and FPG. Although the TyG index showed a reduction in effect size and statistical significance, it was still the major determinant of being NGT-1h-high compared with FPG (the TyG index: OR = 1.327, CI 95% 1.03–1.72, *p* = 0.032; FPG: OR = 1.049, CI 95% 1.03–1.06, *p* < 0.001). Notably, the TyG index remained significantly associated with the NGT-1h-high risk even after adding the presence of hypertension, dyslipidemia, and relative therapies to the statistical models.

**Figure 1:**
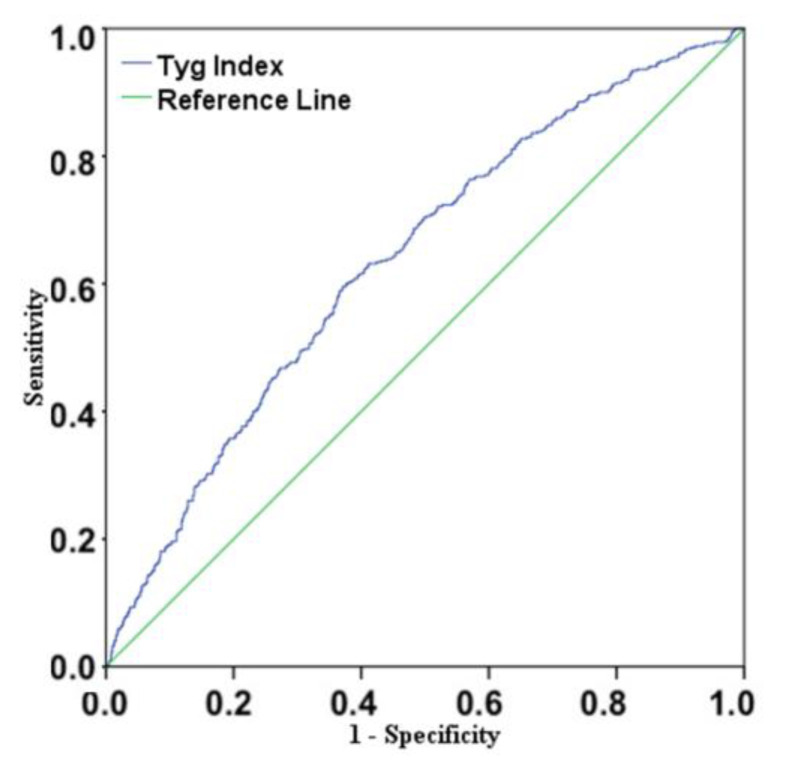
Receiver–operating characteristic (ROC) curve for the accuracy of the TyG index at discriminating NGT-1h-high individuals from NGT-1h-low ones. The accuracy of the TyG index was 61.5% (AUC = 0.615, *p* < 0.001).

The ROC analysis demonstrated that the accuracy of the TyG index for identifying NGT-1h-high patients was 61.5% (AUC = 0.615, CI 95% 0.588–0.643, *p* < 0.001, Figure 1) and that the optimal TyG index cutoff value for discriminating patients with this alteration from those without it was 8.546, a threshold providing 60% sensitivity, 62.4% specificity, a positive predictive value of 42%, and a negative predictive value of 76%.

To promote the use of the TyG index, we generated a simple spreadsheet calculator for the present manuscript (Supplementary File) in which we also embedded a visual indicator that shows if the TyG index values fell above or below our suggested cutoff.

## 4. Discussion

Increasing evidence has suggested that a 1h-OGTT plasma glucose value ≥ 155 mg/dL (8.6 mmol/L) in individuals with NGT is correlated with a higher risk of progression to diabetes. It is also now known that these individuals are more at risk to develop micro- and macrovascular complications or subclinical organ damage, such as common carotid artery thickness [28], left ventricular hypertrophy [7], vascular stiffness [12], and left ventricular diastolic dysfunction [6]; all these factors are independent predictors of cardiovascular events. Consistent with this, two longitudinal studies have shown that 1h-OGTT glucose levels ≥ 161 mg/dL and ≥155 mg/dL predict cardiovascular mortality and all-cause mortality, respectively [15,29].

This increased cardiovascular risk among individuals with dysglycemic conditions is aggravated by the presence of dyslipidemia, a common finding in this kind of patient. In the literature, there is new evidence suggesting that individuals with 1h-OGTT plasma glucose values ≥ 155 mg/dL have a highly atherogenic lipid profile, very similar to that of patients with prediabetes or T2DM [30]. 

Indeed, it has been demonstrated that NGT-1h-high individuals are characterized by skeletal muscle insulin resistance, as confirmed by euglycemic hyperinsulinemic clamp studies and OGTT-derived indexes of insulin sensitivity and reduced insulin clearance; all these elements lead to sustained hyperinsulinemia after an oral glucose load [26,28,31,32]. Impaired insulin sensitivity in the skeletal muscle may divert ingested carbohydrates to the liver instead of muscle glycogen storage. This shift, associated with elevated plasma insulin levels, may lead to increased hepatic de novo lipogenesis. Additionally, hepatic insulin resistance reduces VLDL synthesis and apoB lipoprotein catabolism, resulting in an atherogenic lipid pattern characterized by increased triglyceride concentrations, as observed in NGT-1h-high individuals [33].

Several studies have shown that non-insulin-based indices of insulin resistance are a valid surrogate for the early identification of insulin resistance [17,18]. We focused our investigational work on the TyG index, as many others have evaluated the ability of the TyG index to predict the development of diabetes and prediabetes in patients without dysglycemic conditions [20,21] and with different ethnic backgrounds [20,34,35,36]. The TyG index was shown to possess high accuracy compared with the gold standard hyperinsulinemic–euglycemic clamp; furthermore, evidence has accumulated suggesting that the TyG index may be equal or even superior to the HOMA index in estimating the insulin resistance condition [17,22].

These observations, paired with the accessibility of an accurately characterized cohort of non-diabetic patients involved in the CATAMERI study, prompted us to assess the relationship between 1h-OGTT plasma glucose ≥ 155 mg/dL and the TyG index. Our hypothesis, in the present cross-sectional study, is that the TyG index could be an important instrument to identify patients with high 1h-OGTT plasma glucose levels, even before conducting an OGTT. 

Our results showed that higher values in the TyG index were significantly associated with the risk of having 1h-OGTT plasma glucose levels ≥ 155 mg/dL (OR = 1.703, CI 95% 1.34–2.17). When comparing the standardized β coefficients estimated by linear regression, the TyG index was less precise at predicting 1h-OGTT glycemic values than FPG. On the other hand, the TyG index behaved better when used to predict which participants fell in the NGT-1h-high group. These observations point to the importance of the “other” component of the TyG index: triglycerides levels. It is widely recognized that FPG is the major predictor of the postprandial glycemic curve shape [37]; participants with similar FPG and higher triglycerides levels have a worse cardiometabolic risk profile, which may manifest also in increased 1h-OGTT levels.

Overall, the peculiarity of the TyG index is that it supplies information about the glycemic and lipid profiles joined as one single value. Several studies have shown evidence of the pancreatic β-cell’s weak antioxidant defense, and oxidative stress has been proved to be an important factor for the pathogenesis of T2DM [38,39,40]. Both glucotoxicity and lipotoxicity can lead to an increased production of reactive oxygen species, causing oxidative stress and β-cell dysfunction, and eventually leading to IR and T2DM [38,39,40]. The long-term exposure to elevated concentrations of free fatty acids is related to the prolonged exposure to TG, resulting in impaired β-cell function [40,41,42,43] and reduced glucose-induced insulin secretion [44,45]. By synthetically representing both aspects, the TyG index may actually have an advantage in predicting a worse cardio-metabolic risk profile than the isolated reading of fasting glucose and triglyceride values. In addition to this, we should remark on the extreme usability of the TyG index, which is simple for calculations and linear interpretations; therefore, it can be easily employed to identify those individuals to be monitored for the development of diabetes.

We estimated that the TyG index had 61.5% accuracy for detecting those individuals whose 1h-OGTT glucose levels were above the risk threshold of 155 mg/dL in a population that would otherwise be considered healthy according to the standard procedures. The negative predictive value of 76% indicated that 76% of individuals whose score was below the cutoff were actually true negatives and did not have 1h-OGTT values ≥ 155 mg/dL. In a real-world clinical context, this information would increase the confidence of the physician that the care supplied is accurate, at the price of a few seconds spent on a dedicated calculator (i.e., the one supplied as Supplementary Material). In difficult or under-resourced areas, this advantage has the potential to save money and improve the quality of life of the referred population. In contexts where gold standard investigative screenings are not accessible (i.e., the office of a general practitioner), consulting the TyG index may help with decision-making processes with the final goal of achieving precision medicine. The TyG index has also the merit of being calculated with variables commonly evaluated in clinical practice that are cheap and readily accessible. Moreover, all the components of the TyG index are modifiable risk factors that respond well to lifestyle modifications such as nutrition and exercise [46]. This means that the TyG index can be efficiently used to monitor throughout time the effectiveness of the strategies employed.

Notably, even if in the literature several studies consider values between four and eight as the normal range of the TyG index [20,47], currently, a cutoff value and stratification of risk linked to dysglycemic conditions are still missing, and the performance that we reported cannot be compared with other studies. Several studies adopting our same formula [48] have indicated similar cutoff values for discriminating the presence of metabolic syndrome [49], metabolic-dysfunction-associated fatty liver disease [50], a risk of cerebrovascular disease [51], and a risk of cardiovascular disease and mortality in the general population [52].

Nonetheless, we advise caution in drawing conclusions; a larger body of confirmatory and exploratory studies either in healthy subjects or in patients with prediabetes is necessary to confirm and elaborate on our hypothesis.

Among the strengths of our study, there was a large sample size encompassing both men and women from a real-world outpatient setting. We collected detailed anthropometric and metabolic variables according to a standardized protocol; biochemical analyses were performed in a single center and the assay of metabolites was realized in fresh blood samples instead of stored samples that may lead to their degradation. Furthermore, we also excluded participants with pharmacological treatments affecting glucose metabolism. 

Caution is advised in the interpretation of our results. The population that we considered was poorly diversified; it was composed of Caucasian individuals residing in the south of Italy, and the generalizability of our results may be hindered in other ethnic groups. Another limitation of our study was that the participants underwent a single 75 g OGTT to evaluate glucose tolerance. Even if this approach is common in both clinical practice and epidemiological studies, the intra-individual variability of 1 h and 2 h post-challenge glucose could not be assessed, possibly leading to some inaccuracies in the classification of the recruited participants into glucose tolerance groups. Moreover, we recruited participants in a referral university hospital, and our findings may not be extendible to the general population. 

Finally, the cross-sectional design of the study reflected only an association of the TyG index with 1h-OGTT plasma glucose and precludes us to draw any conclusion about the causal relationships between this dysglycemic condition and the development of metabolic diseases such as T2DM. This first report on the matter should be considered hypothesis-generating and requiring confirmation by additional prospective studies assessing the impact of the TyG index in predicting the variability of 1h-OGTT plasma glucose values.

## 5. Conclusions

In conclusion, our findings suggest that the TyG index could be a useful tool to screen, evaluate, and, where appropriate, beforehand treat individuals with high 1h-OGTT glucose levels.

## Figures and Tables

**Table 1:** Anthropometric and metabolic characteristics of study subjects stratified according to one hour post load glycaemia.

Variables	Whole Study Group	NGT-1h-Low	NGT-1h-High	*p*
Gender (M/F)	813/661	623/381	190/280	<0.001 *
Age (years)	46 (±13)	43 (±13)	50 (±11)	<0.001 **
BMI (Kg/m^2^)	29.7 (±6.4)	29.5 (±6.5)	30.3 (±6.0)	0.027 ***
SBP (mg/dL)	126.4 (±17.2)	125.1 (±17.4)	129.1 (±16.3)	<0.001
DBP (mg/dL)	79.2 (±11.3)	78.5 (±11.6)	80.7 (±10.5)	0.001
Tot-COL (mg/dL)	198.4 (±38.7)	197.1 (±39.4)	201.3 (±37.0)	0.052
HDL-Col (mg/dL)	52.2 (±13.4)	53.4 (±14.1)	49.7 (±13.3)	<0.001
LDL-Col (mg/dL)	127.2 (±34.2)	125.7 (±34.6)	130.3 (±33.2)	0.017
Triglycerides (mg/dL)	120.2 (±70.2)	113.4 (±68.4)	134.7 (±71.9)	<0.001
AST (UI/dL)	21.4 (±10.2)	20.9 (±10.2)	22.4 (±10.0)	0.008
ALT (UI/dL)	24.3 (±16.3)	23.1 (±14.2)	26.7 (±20.0)	<0.001
gamma-GT (ng/L)	25.0 (±22.9)	23.4 (±22.3)	28.5 (±23.6)	<0.001
Fasting glucose (mg/dL)	90.9 (±9.7)	88.8 (±8.8)	95.2 (±10.3)	<0.001
1-h glucose (mg/dL)	139.0 (±37.2)	118.9 (±23.1)	181.8 (±22.6)	<0.001
2-h glucose (mg/dL)	106.8 (±20.3)	102.6 (±19.5)	115.8 (±18.5)	<0.001
Fasting insulin (mU/mL)	12.9 (±8.6)	12.4 (±8.6)	14.1 (±8.4)	<0.001
HOMA-IR	2.9 (±2.0)	2.7 (±1.9)	3.3 (±2.0)	<0.001
TyG index	8.46 (±0.53)	8.39 (±0.52)	8.64 (±0.51)	<0.001
Smoking habits (Yes/No)	344/1130	224/780	120/350	0.173
Lipid-lowering therapy (Yes/No)	185/1289	86/918	99/371	0.004
Hypertension (Yes/No)	874/600	543/461	331/139	<0.001
Antihypertensive therapy (Yes/No)	667/807	403/601	264/206	<0.001

The data are presented as means ± SD for continuous variables and number for dichotomous variables. Comparisons were performed using a general linear model with post hoc Bonferroni correction for multiple comparisons and with the g^2^ test for categorical variables. *p* values refer to results after analyses with adjustment for age, gender, and BMI. * *p* values refer to results after analyses with adjustment for age and BMI. ** *p* values refer to results after analyses with adjustment for gender and BMI. *** *p* values refer to results after analyses with adjustment for age and gender. BMI = body mass index; SBP = systolic blood pressure; DBP = diastolic blood pressure; Tot-COL = total cholesterol; HDL-Col = high-density lipoprotein; LDL-Col = low-density lipoprotein; AST = aspartate aminotransferase; ALT = alanine transaminase; gamma-GT = gamma-glutamyl transpeptidase; HOMA-IR = homeostasis-model-assessment-estimated insulin resistance; the TyG index = triglycerides and glucose index.

**Table 2:** Stepwise multivariable regression analysis of 1 h-OGTT as dependent variable and different covariates in the whole population.

Dependent Variable 1h-OGTT	Independent Contributors	Standardized Coefficient	*p*
**Model 1**	**Gender**	0.155	<0.001
	**Age**	0.300	<0.001
	**BMI**	0.109	<0.001
	**TyG Index**	0.191	<0.001
**Model 2**	**Gender**	0.157	<0.001
	**Age**	0.246	<0.001
	**BMI**	0.125	0.005
	**FPG**	0.284	<0.001
**Model 3**	**Gender**	0.189	<0.001
	**Age**	0.351	<0.001
	**BMI**	0.076	0.004
	**HOMA-IR**	0.159	<0.001
**Model 4**	**Gender**	0.133	<0.001
	**Age**	0.231	<0.001
	**BMI**	0.103	0.017
	**TyG Index**	0.115	0.032
	**FPG**	0.249	<0.001

OGTT = oral glucose tolerance test; BMI = body mass index; the TyG index = triglyceride glucose index; HOMA-IR = homeostatic model assessment for insulin resistance; FPG = fasting plasma glucose.

**Table 3:** Multiple logistic regression analysis for the NGT 1 h-high risk.

NGT-1h-High Risk	Variable	OR	95% CI	*p*
**Model 1**	**Gender**	2.071	1.63–2.64	<0.001
	**Age**	1.042	1.03–1.05	0.045
	**BMI**	1.026	1.01–1.05	0.010
	**TyG Index**	1.703	1.34–2.17	<0.001
**Model 2**	**Gender**	2.055	1.62–2.61	<0.001
	**Age**	1.034	1.02–1.04	<0.001
	**BMI**	1.028	1.00–1.05	0.005
	**FPG**	1.054	1.04–1.07	<0.001
**Model 3**	**Gender**	2.232	1.76–2.82	<0.001
	**Age**	1.047	1.04–1.06	<0.001
	**BMI**	1.011	0.99–1.03	0.318
	**HOMA-IR**	1.156	1.08–1.23	<0.001
**Model 4**	**Gender**	1.942	1.52–2.48	<0.001
	**Age**	1.033	1.02–1.04	<0.001
	**BMI**	1.024	1.01–1.04	0.017
	**TyG Index**	1.327	1.03–1.72	0.032
	**FPG**	1.049	1.03–1.06	<0.001

NGT = normal glucose tolerance; BMI = body mass index; the TyG index = triglyceride glucose index; HOMA-IR = homeostatic model assessment for insulin resistance; FPG = fasting plasma glucose.

## Data Availability

All data are contained within the article or Supplementary Materials.

## Supplementary Materials

The following supporting information can be downloaded at: https://www.mdpi.com/article/10.3390/ijerph20010787/s1.

## References

[1] Kerner W., Brückel J., German Diabetes Association (2014). Definition, classification and diagnosis of diabetes mellitus. Exp. Clin. Endocrinol. Diabetes Off. J. Ger. Soc. Endocrinol. Ger. Diabetes Assoc..

[2] Sun H., Saeedi P., Karuranga S., Pinkepank M., Ogurtsova K., Duncan B.B., Stein C., Basit A., Chan J.C., Mbanya J.C. (2022). IDF Diabetes Atlas: Global, regional and country-level diabetes prevalence estimates for 2021 and projections for 2045. Diabetes Res. Clin. Pract..

[3] Galaviz K.I., Narayan K.M.V., Lobelo F., Weber M.B. (2015). Lifestyle and the Prevention of Type 2 Diabetes: A Status Report. Am. J. Lifestyle Med..

[4] Knowler W.C., Barrett-Connor E., Fowler S.E., Hamman R.F., Lachin J.M., Walker E.A., Nathan D.M., Diabetes Prevention Program Research Group (2002). Reduction in the incidence of type 2 diabetes with lifestyle intervention or metformin. N. Engl. J. Med..

[5] Alyass A., Almgren P., Akerlund M., Dushoff J., Isomaa B., Nilsson P., Tuomi T., Lyssenko V., Groop L., Meyre D. (2015). Modelling of OGTT curve identifies 1 h plasma glucose level as a strong predictor of incident type 2 diabetes: Results from two prospective cohorts. Diabetologia.

[6] Priya M., Anjana R.M., Chiwanga F.S., Gokulakrishnan K., Deepa M., Mohan V. (2013). 1-hour venous plasma glucose and incident prediabetes and diabetes in Asian indians. Diabetes Technol. Ther..

[7] Abdul-Ghani M.A., Abdul-Ghani T., Ali N., Defronzo R.A. (2008). One-hour plasma glucose concentration and the metabolic syndrome identify subjects at high risk for future type 2 diabetes. Diabetes Care.

[8] Jagannathan R., Sevick M.A., Fink D., Dankner R., Chetrit A., Roth J., Buysschaert M., Bergman M. (2016). The 1-hour post-load glucose level is more effective than HbA1c for screening dysglycemia. Acta Diabetol..

[9] Bergman M., Abdul-Ghani M., DeFronzo R.A., Manco M., Sesti G., Fiorentino T.V., Ceriello A., Rhee M., Phillips L.S., Chung S. (2020). Review of methods for detecting glycemic disorders. Diabetes Res. Clin. Pract..

[10] Bergman M., Manco M., Sesti G., Dankner R., Pareek M., Jagannathan R., Chetrit A., Abdul-Ghani M., Buysschaert M., Olsen M.H. (2018). Petition to replace current OGTT criteria for diagnosing prediabetes with the 1-hour post-load plasma glucose ≥ 155 mg/dL (8.6 mmol/L). Diabetes Res. Clin. Pract..

[11] Fiorentino T.V., Marini M.A., Succurro E., Andreozzi F., Perticone M., Hribal M.L., Sciacqua A., Perticone F., Sesti G. (2018). One-Hour Postload Hyperglycemia: Implications for Prediction and Prevention of Type 2 Diabetes. J. Clin. Endocrinol. Metab..

[12] Fiorentino T.V., Marini M.A., Andreozzi F., Arturi F., Succurro E., Perticone M., Sciacqua A., Hribal M.L., Perticone F., Sesti G. (2015). One-Hour Postload Hyperglycemia Is a Stronger Predictor of Type 2 Diabetes Than Impaired Fasting Glucose. J. Clin. Endocrinol. Metab..

[13] Jagannathan R., Sevick M.A., Li H., Fink D., Dankner R., Chetrit A., Roth J., Bergman M. (2016). Elevated 1-hour plasma glucose levels are associated with dysglycemia, impaired beta-cell function, and insulin sensitivity: A pilot study from a real world health care setting. Endocrine.

[14] Pareek M., Bhatt D.L., Nielsen M.L., Jagannathan R., Eriksson K.-F., Nilsson P.M., Bergman M., Olsen M.H. (2018). Enhanced Predictive Capability of a 1-Hour Oral Glucose Tolerance Test: A Prospective Population-Based Cohort Study. Diabetes Care.

[15] Strandberg T.E., Pienimäki T., Salomaa V.V., Pitkala K.H., Tilvis R.S., Miettinen T.A. (2011). One-hour glucose, mortality, and risk of diabetes: A 44-year prospective study in men. Arch. Intern. Med..

[16] Saunajoki A., Auvinen J., Bloigu A., Ukkola O., Keinänen-Kiukaanniemi S., Timonen M. (2021). One-hour post-load glucose improves the prediction of cardiovascular events in the OPERA study. Ann. Med..

[17] Guerrero-Romero F., Simental-Mendía L.E., González-Ortiz M., Martínez-Abundis E., Ramos-Zavala M.G., Hernández-González S.O., Jacques-Camarena O., Rodríguez-Morán M. (2010). The product of triglycerides and glucose, a simple measure of insulin sensitivity. Comparison with the euglycemic-hyperinsulinemic clamp. J. Clin. Endocrinol. Metab..

[18] Martin B., Warram J., Krolewski A., Soeldner J., Kahn C., Bergman R. (1992). Role of glucose and insulin resistance in development of type 2 diabetes mellitus: Results of a 25-year follow-up study. Lancet Lond. Engl..

[19] Jin J.-L., Cao Y.-X., Wu L.-G., You X.-D., Guo Y.-L., Wu N.-Q., Zhu C.-G., Gao Y., Dong Q.-T., Zhang H.-W. (2018). Triglyceride glucose index for predicting cardiovascular outcomes in patients with coronary artery disease. J. Thorac. Dis..

[20] Navarro-González D., Sánchez-Íñigo L., Pastrana-Delgado J., Fernández-Montero A., Martinez J.A. (2016). Triglyceride-glucose index (TyG index) in comparison with fasting plasma glucose improved diabetes prediction in patients with normal fasting glucose: The Vascular-Metabolic CUN cohort. Prev. Med..

[21] Wen J., Wang A., Liu G., Wang M., Zuo Y., Li W., Zhai Q., Mu Y., Gaisano H.Y., He Y. (2020). Elevated triglyceride-glucose (TyG) index predicts incidence of Prediabetes: A prospective cohort study in China. Lipids Health Dis..

[22] Vasques A.C.J., Novaes F.S., de Oliveira M.D.S., Souza J.R.M., Yamanaka A., Pareja J.C., Tambascia M.A., Saad M.J.A., Geloneze B. (2011). TyG index performs better than HOMA in a Brazilian population: A hyperglycemic clamp validated study. Diabetes Res. Clin. Pract..

[23] Andreozzi F., Succurro E., Mancuso M.R., Perticone M., Sciacqua A., Perticone F., Sesti G. (2007). Metabolic and cardiovascular risk factors in subjects with impaired fasting glucose: The 100 versus 110 mg/dL threshold. Diabetes Metab. Res. Rev..

[24] Marini M.A., Succurro E., Frontoni S., Hribal M.L., Andreozzi F., Lauro R., Perticone F., Sesti G. (2007). Metabolically healthy but obese women have an intermediate cardiovascular risk profile between healthy nonobese women and obese insulin-resistant women. Diabetes Care.

[25] (1980). WHO Expert Committee on Diabetes Mellitus: Second report. World Health Organ. Tech. Rep. Ser..

[26] Sesti G., Hribal M.L., Fiorentino T.V., Sciacqua A., Perticone F. (2014). Elevated 1 h postload plasma glucose levels identify adults with normal glucose tolerance but increased risk of non-alcoholic fatty liver disease. BMJ Open Diabetes Res. Care.

[27] Park K., Ahn C.W., Lee S.B., Kang S., Nam J.S., Lee B.K., Kim J.H., Park J.S. (2019). Elevated TyG Predicts Progression of Coronary Artery Calcification. Diabetes Care.

[28] Iraj B., Salami R., Feizi A., Amini M. (2015). The profile of hypertension and dyslipidemia in prediabetic subjects; results of the Isfahan Diabetes Prevention program: A large population-based study. Adv. Biomed. Res..

[29] Bergman M., Chetrit A., Roth J., Dankner R. (2016). One-hour post-load plasma glucose level during the OGTT predicts mortality: Observations from the Israel Study of Glucose Intolerance, Obesity and Hypertension. Diabet. Med. J. Br. Diabet. Assoc..

[30] Andreozzi F., Mannino G.C., Perticone M., Perticone F., Sesti G. (2017). Elevated 1-h post-load plasma glucose levels in subjects with normal glucose tolerance are associated with a pro-atherogenic lipid profile. Atherosclerosis.

[31] Marini M.A., Succurro E., Frontoni S., Mastroianni S., Arturi F., Sciacqua A., Lauro R., Hribal M.L., Perticone F., Sesti G. (2012). Insulin sensitivity, β-cell function, and incretin effect in individuals with elevated 1-hour postload plasma glucose levels. Diabetes Care.

[32] Marini M.A., Frontoni S., Succurro E., Arturi F., Fiorentino T.V., Sciacqua A., Hribal M.L., Perticone F., Sesti G. (2013). Decreased insulin clearance in individuals with elevated 1-h post-load plasma glucose levels. PloS ONE.

[33] Marini M., Succurro E., Arturi F., Ruffo M., Andreozzi F., Sciacqua A., Lauro R., Hribal M., Perticone F., Sesti G. (2012). Comparison of A1C, fasting and 2-h post-load plasma glucose criteria to diagnose diabetes in Italian Caucasians. Nutr. Metab. Cardiovasc. Dis. NMCD.

[34] Lee S.-H., Kwon H.-S., Park Y.-M., Ha H.-S., Jeong S.H., Yang H.K., Lee J.-H., Yim H.-W., Kang M.-I., Lee W.-C. (2014). Predicting the development of diabetes using the product of triglycerides and glucose: The Chungju Metabolic Disease Cohort (CMC) study. PLoS ONE.

[35] Lee J.-W., Lim N.-K., Park H.-Y. (2018). The product of fasting plasma glucose and triglycerides improves risk prediction of type 2 diabetes in middle-aged Koreans. BMC Endocr. Disord..

[36] Low S., Khoo K.C.J., Irwan B., Sum C.F., Subramaniam T., Lim S.C., Wong T.K.M. (2018). The role of triglyceride glucose index in development of Type 2 diabetes mellitus. Diabetes Res. Clin. Pract..

[37] Petersen K.F., Dufour S., Savage D.B., Bilz S., Solomon G., Yonemitsu S., Cline G.W., Befroy D., Zemany L., Kahn B.B. (2007). The role of skeletal muscle insulin resistance in the pathogenesis of the metabolic syndrome. Proc. Natl. Acad. Sci. USA.

[38] Robertson R.P., Harmon J., Tran P.O.T., Poitout V. (2004). Beta-cell glucose toxicity, lipotoxicity, and chronic oxidative stress in type 2 diabetes. Diabetes.

[39] Robertson R.P., Harmon J., Tran P.O., Tanaka Y., Takahashi H. (2003). Glucose toxicity in beta-cells: Type 2 diabetes, good radicals gone bad, and the glutathione connection. Diabetes.

[40] Robertson R., Zhou H., Zhang T., Harmon J.S. (2007). Chronic oxidative stress as a mechanism for glucose toxicity of the beta cell in type 2 diabetes. Cell Biochem. Biophys..

[41] Mason T.M., Goh T., Tchipashvili V., Sandhu H., Gupta N., Lewis G.F., Giacca A. (1999). Prolonged elevation of plasma free fatty acids desensitizes the insulin secretory response to glucose in vivo in rats. Diabetes.

[42] Jacqueminet S., Briaud I., Rouault C., Reach G., Poitout V. (2000). Inhibition of insulin gene expression by long-term exposure of pancreatic beta cells to palmitate is dependent on the presence of a stimulatory glucose concentration. Metabolism.

[43] Maedler K., Spinas G., Dyntar D., Moritz W., Kaiser N., Donath M.Y. (2001). Distinct effects of saturated and monounsaturated fatty acids on beta-cell turnover and function. Diabetes.

[44] Zhou Y.P., Grill V.E. (1994). Long-term exposure of rat pancreatic islets to fatty acids inhibits glucose-induced insulin secretion and biosynthesis through a glucose fatty acid cycle. J. Clin. Investig..

[45] Zhou Y.P., Grill V. (1995). Long term exposure to fatty acids and ketones inhibits B-cell functions in human pancreatic islets of Langerhans. J. Clin. Endocrinol. Metab..

[46] Schellenberg E.S., Dryden D.M., Vandermeer B., Ha C., Korownyk C. (2013). Lifestyle interventions for patients with and at risk for type 2 diabetes: A systematic review and meta-analysis. Ann. Intern. Med..

[47] Tripathy D., Almgren P., Tuomi T., Groop L. (2004). Contribution of insulin-stimulated glucose uptake and basal hepatic insulin sensitivity to surrogate measures of insulin sensitivity. Diabetes Care.

[48] Alizargar J., Hsieh N.C., Wu S.V. (2020). The correct formula to calculate triglyceride-glucose index (TyG). J. Pediatr. Endocrinol. Metab..

[49] Nabipoorashrafi S.A., Seyedi S.A., Rabizadeh S., Ebrahimi M., Ranjbar S.A., Reyhan S.K., Meysamie A., Nakhjavani M., Esteghamati A. (2022). The accuracy of triglyceride-glucose (TyG) index for the screening of metabolic syndrome in adults: A systematic review and meta-analysis. Nutr. Metab. Cardiovasc. Dis..

[50] Wang J., Yan S., Cui Y., Chen F., Piao M., Cui W. (2022). The Diagnostic and Prognostic Value of the Triglyceride-Glucose Index in Metabolic Dysfunction-Associated Fatty Liver Disease (MAFLD): A Systematic Review and Meta-Analysis. Nutrients.

[51] Yan F., Yan S., Wang J., Cui Y., Chen F., Fang F., Cui W. (2022). Association between triglyceride glucose index and risk of cerebrovascular disease: Systematic review and meta-analysis. Cardiovasc. Diabetol..

[52] Liu X., Tan Z., Huang Y., Zhao H., Liu M., Yu P., Ma J., Zhao Y., Zhu W., Wang J. (2022). Relationship between the triglyceride-glucose index and risk of cardiovascular diseases and mortality in the general population: A systematic review and meta-analysis. Cardiovasc. Diabetol..

